# Management of septic arthritis of the hip joint in adults. A systematic review of the literature

**DOI:** 10.1186/s12891-021-04843-z

**Published:** 2021-12-02

**Authors:** Giovanni Balato, Vincenzo de Matteo, Tiziana Ascione, Roberto de Giovanni, Ernesto Marano, Maria Rizzo, Massimo Mariconda

**Affiliations:** 1 Department of Public Health, Orthopedic Unit, “Federico II” University, Via Sergio Pansini, 5, 80130 Naples, Italy; 2 Service of Infectious Diseases, AORN Cardarelli Hospital, Naples, Italy

**Keywords:** Septic arthritis, Hip joint, Adult, Infection

## Abstract

**Background:**

The septic arthritis of the hip is a complex condition characterized by a variety of clinical presentations, a challenging diagnosis and different surgical treatment options, including arthroscopy, resection arthroplasty and one and two-stage total hip replacement. Each technique reports variable results in terms of infection eradication rate. The aim of this systematic review is to compare the most relevant studies available in current literature and to assess if a better treatment outcome can be predicted based on the microbiology, history, and type of infection (active vs quiescent) of each case.

**Methods:**

A systematic review of the literature was performed in accordance with the PRISMA guidelines, including the studies dealing with the treatment of hip septic arthritis in adult patients. Electronic databases, namely the MEDLINE, Scopus, and Web of Science, were reviewed using a combination of following keywords “septic arthritis” AND “hip joint” OR “hip” AND “adult”.

**Results:**

The total number of patients included in this review was 1236 (45% of which females), for 1238 hips. The most common pathogen isolated was *Staphylococcus aureus* in its Methicillin-sensitive variant ranging from 2 to 37% of cases. Negative cultures were the second most common finding. It was also differentiated the type of infection of the hip, 809 and 417 patients with active and quiescent hip infection, respectively, were analyzed. Eradication rates for two-stage revision arthroplasty ranged between 85 and 100%, for one-stage approach between 94 and 100%, while for arthroscopic debridement/lavage between 89 and 100%.

**Conclusion:**

*Staphylococcus aureus* is the most common microorganism isolated followed by culture negative infections. Arthroscopic, one and two stage procedures can be effective in the treatment of hip septic arthritis when the indication is consistent with the type of infection retrieved.

**Level of evidence:**

IV, therapeutic study.

## Background

Septic Arthritis (SA) of native adult hip represents an uncommon but severe condition with possible sequelae including accelerated joint degeneration, osteonecrosis, disability and with an estimated mortality rate of 11% [1–3].

Due to the possible clinical presentations, which may vary based on age [4], type of infection and etiology, the diagnostic workup and definitive treatment require a multidisciplinary approach. A timely diagnosis is essential in order to avoid a delayed treatment which could result in quality life-altering consequences for the patient [5]. Furthermore, several algorithms tried to standardize the diagnostic procedures and treatment of septic arthritis, but no consensus has been reached so far, probably due to the small number of patients included in the studies available.

Various surgical treatment options are currently available for the orthopedic surgeon facing a SA including arthroscopic lavage/debridement, resection arthroplasty (arthrotomy) and Total Hip replacement (THR) in one or two stages [6, 7]. The Second International Consensus Meeting (ICM) on orthopedic infections in 2018 tried to standardize the treatment of the patient with SA differentiating between active and quiescent local infective process of the hip or knee [8]. Patients with quiescent infection often reported a distant history of infections and the clinical and laboratory investigations including serum, synovial aspirate and imaging studies demonstrated no symptoms and signs of active infections. Recently, a systematic review by D’Angelo et al. found that arthroscopy, single open or two-stage THA are effective in treating bacterial septic arthritis of the native hip [9]. Since then, some additional studies have assessed the treatment outcomes of septic arthritis of native adult hip. Therefore, we carried out an updated systematic literature review to further address the success rate and outcome of patients affected by hip SA surgically treated.

## Methods

### Search strategy and criteria

This systematic review was conducted according to the guidelines of the Preferred Reporting Items for Systematic Review and Meta-Analyses (PRISMA )[10]. Electronic databases, namely the MEDLINE, Scopus, and Web of Science, were reviewed for studies investigating the treatment of hip septic arthritis in adult patients. A combination of following keywords was used for article search: “Septic arthritis” AND “hip joint” OR “hip” AND “adult”. The inclusion criteria were not limited to English language literature and specific publication dates. Reference lists of selected articles were searched for any additional articles that were not identified in the database search. Longitudinal studies (retrospective and prospective) evaluating patients affected by hip septic arthritis surgically treated were included. The exclusion criteria included: case reports, expert opinions, prior systematic reviews, letters to the editor and studies that included different joints involved in which hip data could not be extrapolated.

### Study assessment and data extraction

Initially, the titles and abstracts of the studies were screened by two pairs of independent reviewers (RdG, EM). Full text was obtained for all the abstracts that appeared to meet the inclusion criteria or those with any uncertainty. Then, each study was assessed based on the inclusion criteria by two independent reviewers and any disagreement regarding inclusion of any particular study was resolved by evaluation of the article by the senior author (GB).

**Fig. 1:**
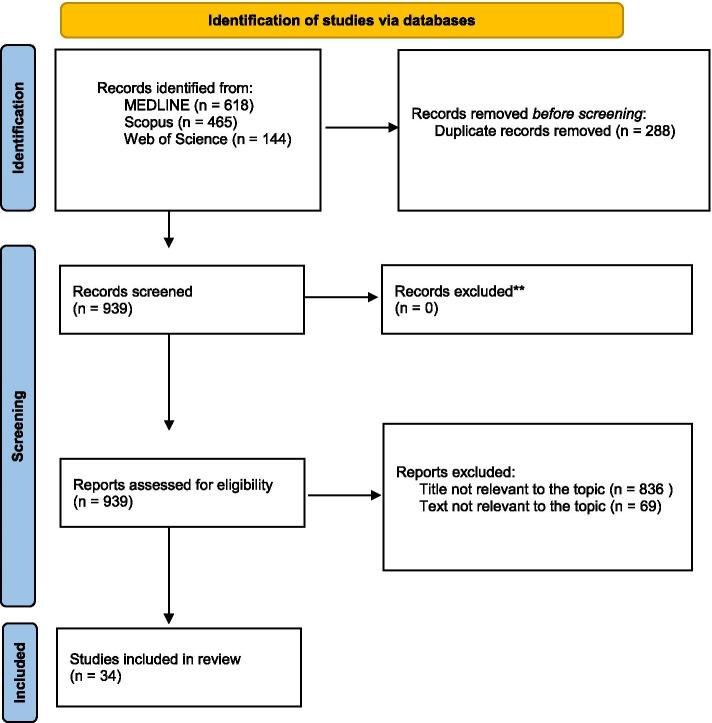
Search strategy

The flow diagram of our search strategy is presented in Fig. 1. A total of 1227 potentially relevant studies were found through computer search and manual screening of reference lists; 288 were duplicates and were removed. After screening the titles and abstracts, 836 studies were excluded, and 103 full texts were evaluated. 69 studies were excluded after a detailed assessment and the remaining 34 articles were included in our systematic review [1, 3–5, 11–40].

Relevant data were extracted from each included study. Data describing participants demographics, microbiology, treatment options and outcomes were recorded.

## Results

### Patient analyzed

Table 1 summarizes the characteristic of the included studies. A total of 1236 patients (1238 hips) affected by septic arthritis were evaluated. Based on reported data, hip infection occurs in patients with a mean age ranging from 24 to 65 years. Of 1116 patients, 45% were female [1, 4, 11, 13, 16–22, 25–33, 35, 36, 38–41]. All studies included in our systematic review clearly described the type of infection distinguishing the septic process in active or quiescent as established during the second International Consensus Meeting in Philadelphia [8]. Active infection is defined as the presence of clinical and laboratory findings of local infection while a quiescent infection refers to an history of septic arthritis with no signs of active infection. We included 809 and 417 patients with active and quiescent hip infection, respectively. The mean follow-up was reported in all studies included ranging from 3.3 months [25] to 182.4 months [4].

**Table 1:** Characteristics of the studies included in the systematic review

First author, year, Nation	Journal	Study design	N patients	Age (years)	Sex	Infection Classification (Active vs. Quiescent)	Average Follow-up Duration (Months)
Anagnostakos et al. 2016 [11] *(Germany)*	Archives of Orthopaedic and Trauma Surgery	Retrospective	22	59.7	11 M 11 F	Active	44.8
Bauer et al. 2010 [12] *(France)*	Orthopaedics & Traumatology: Surgery & Research	Retrospective	22	60	N.A.	Active and quiescent	60
Chen et al. 2008 [13] *(China)*	International Orthopaedics	Retrospective	28	53	22 M 6 F	Active	77
Cho et al. 2018 [14] *(South Korea)*	The Journal of Arthroplasty	Retrospective	10	44.6	5 M 4 F	Active	44.9
Choe et al. 2015 [15] *(Japan)*	Modern Rheumatology	Retrospective	27	65	N.A.	Active	33
El Ganzoury et al. 2015 [16] *(Egypt)*	Journal of Orthopaedics	Prospective	23	45	15 M 8 F	Active	48
Ferrand et al. 2016 [17] *(France)*	Infectious Diseases	Ambispective	12	60.1	74 M 35 F	Active	17
Fleck et al. 2011 [18] *(USA)*	Clinical Orthopaedics and Related Research	Retrospective	14	60.8	M 7 F 7	Active	50
Flores-Robles et al. 2019 [3] *(Spain)*	Journal of Clinical Rheumatology	Retrospective	7	56	N.A.	Active	12
Fukushima et al. 2021 [19] *(Japan)*	BMC Musculoskeletal Disorders	Retrospective	5	46.2	M 5	Active	40.2
Gao et al. 2010 [20] *(China)*	Chinese Medical Journal	Retrospective	19	40.7	7 M 12 F	Quiescent	34
Huang et al. 2010 [26] *(Taiwan)*	Journal of Trauma and Acute Care Surgery	Retrospective	14	54.3	M 9 F 5	Active	42.5
Hunter et al. 2015 [5] *(USA)*	Journal of bone and joint surgery	Retrospective	3	55.5	N.A.	Active	9 (if one stage was successful).4.9 for single- surgery failure
Kaminski et al. 2007 [21] *(Germany)*	Ortopediia Traumatologia Rehabilitacja	Retrospective	5	29.4	4 M 1 F	Active	52
Kao et al. 2019 [1] *(Taiwan)*	Medicine (Baltimore)	Retrospective	51	58.7	M 32 F 19	Active	48.8
Khazi et al. 2020 [22] *(USA)*	Arthroscopy	Retrospective	421	N.A.	222 M 199 F	Active	1
Kim et al. 2003 [23] *(South Korea)*	Journal of Bone and Joint Surgery	Retrospective	170	42.3	N.A.	Quiescent	119
Kim et al. 2009 [4] *(South Korea)*	Clinical Orthopaedics and Related Research	Retrospective	62	47.5	22 M 40 F	Quiescent	182.4
Kim et al. 2018 [24] *(South Korea)*	Hip International	Retrospective	7	50.9	M 4 F 3	Active	16
Kunze et al. 2020 [25] *(USA)*	Arthroplasty today	Retrospective	12	60.2	M 7 F 5	Active and quiescent	3.3
Lee et al. 2014 [27] *(South Korea)*	Knee Surgery, Sports, traumatology, Arthroscopy	Retrospective	9	45	M 4 F 5	Active	18
Li et al. 2016 [28] *(China)*	Journal of Orthopaedic Surgery and Research	Retrospective	9	50	M 5 F 4	Active	40
Lustig et al. 2007 [29] *(France)*	Revue de chirurgie orthopedique et reparatrice de l’appareil moteur	Retrospective	17	53	6 M 11 F	Quiescent	72
Nusem et al. 2006 [30] *(Australia)*	Arthroscopy	Retrospective	6	24	M 3 F 3	Active	22
Ohtsuru et al. 2016 [31] *(Japan)*	Hip International	Retrospective	15	55.9	9 M 6 F	Active	N.A.
Papanna et al. 2017 [32] *(Japan)*	Hip International	Retrospective	18	58	M 21 F 15	Active and quiescent	70 72
Park et al. 2005 [33] *(South Korea)*	The Journal of Arthroplasty	Retrospective	75	51.8	36 M 39 F	Quiescent	70
Romanò et al. 2012 [41] *(Italy)*	BMC Infectious Diseases	Prospective	19	55.7	M 9 F 10	Quiescent	56.6
Russo et al. 2021 [35] *(Italy)*	International Orthopaedics	Retrospective	25	56.4	M 13 F 12	Active	85.2
Schroder et al. 2016 [36] *(Germany)*	Advances in Orthopedics	Retrospective	7	44	M 4 F 3	Active	27
Shen et al. 2013 [37] *(China)*	Orthopedics	Retrospective	5	40	N.A.	Active	40
Xu et al. 2019 [38] *(China)*	BMC Musculoskeletal Disorders	Retrospective	55	45.8	M 41 F 14	Active	62
Yamamoto et al. 2001 [39] *(Japan)*	Arthroscopy	Retrospective	4	59	M 1 F 3	Active	32
Yoo et al. 2009 [40] *(South Korea)*	Clinical Orthopaedics and Related Research	Retrospective	38	44	13 M 25 F	Quiescent	100

*N.A.* Not Available

### Etiology and pathogenesis

All but six [17,20,22,23,37,40] of the included studies clearly stated the pathogens responsible for the hip SA (Table 2). In each of these studies the species *Staphylococcus aureus* was the most common finding, with the exception of the study by Li et al. [28], in which all the presented hip SA were sustained by *Mycobacterium tuberculosis*, and 4 studies in which the majority of patients had negative culture infections. Methicillin-sensitive *Staphylococcus aureus* (MSSA) was responsible for SA in a percentage of patients that varied from 2 to 37% [3,11,14,18,24,30,32,35,36,39,41], while 3 found an higher isolation rate for Methicillin Resistant *S. aureus* (MRSA) [13,15,31].

**Table 2:** Microbiological findings and the cause of hip septic arthritis

First author, year, Nation	Pathogens	Cause of infection
Anagnostakos et al. 2016 [11] *(Germany)*	*MSSA (72.7%)* Negative culture (27.3%)	N.A.
Bauer et al. 2010 [12] *(France)*	*MSSA (40.9%)* *Coagulase-negative Staphylococcus (27.3%)* *Streptococcus species (13.6%)* Gram - bacteria (not specified) (9.1%) Polymicrobial (9.1%)	Post-operative (54.5%) Hematogenous (45.5%)
Chen et al. 2008 [13] *(China)*	*MRSA (28.6%)* *MSSA (21.4%)* *Salmonella species (10.7%)* *Escherichia coli (10.7%)* *Pseudomonas aeruginosa (3.6%)* *viridans Streptococcus (3.6%)* *Prevotella melaninogenica (3.6%)* *Enterococcus species (3.6%)* *Enterobacter cloacae (3.6%)* Polymicrobial (10.7%)	N.A.
Cho et al. 2018 [14] *(South Korea)*	*MSSA (40.0%)* Other (20.0%) Negative culture (40.0%)	N.A.
Choe et al. 2015 [15] *(Japan)*	*MRSA (37%)* *MSSA (7.4%)* *Streptococcus agalactiae (3.7%)* *Escherichia coli (3.7%)* *Staphylococcus epidermidis (3.7%)* *Bacillus (not specified) (3.7%)* *Pseudomonas aeruginosa (3.7%)* *Enterococcus faecalis (3.7%)* Negative culture (29.6%)	N.A.
El Ganzoury et al. 2015 [16] *(Egypt)*	*MSSA (48%);* *Staphylococcus epidermidis (30%)*	N.A.
Ferrand et al. 2016 [17] *(France)*	N.A.	N.A.
Fleck et al. 2011 [18] *(USA)*	*MSSA (35.7%)* *MRSA (21.4%)* Other (not specified) (21.4%) Negative culture (21.4%)	Hematogenous (64.3%) After local injection (14.3%) Post-traumatic (21.4%)
Flores-Robles et al. 2019 [3] *(Spain)*	*MSSA (39.7%)* *MRSA (6.4%)* *Staphylococcus capitis (4.8%)* *Staphylococcus epidermidis (1.6%)* *Streptococcus mitis (3.2%)* *Streptococcus milleri (1.6%)* *Streptococcus oralis (1.6%)* *Streptococcus agalactiae (4.8%)* *Streptococcus pneumoniae (1.6%)* *Enterococcus faecalis (1.6%)* *Escherichia coli (3.2%)* *Fusobacterium nucleatum (1.6%)* *Nocardia cyriacigeorgica (1.6%)* *Eikenella corrodens (3.2%)* Negative culture (23.8%)	Hematogenous (65.3%) Infiltration (12%) Catheter (6%)
Fukushima et al. 2021 [19] *(Japan)*	*Staphylococcus species (not specified) (20%)* *Streptococcus agalactiae (Group B) (20%)* *Haemophilus influenzae (20%)* *MSSA (20%)* Negative culture (20%)	N.A.
Gao et al. 2010 [20] *(China)*	N.A.	N.A.
Huang et al. 2010 [26] *(Taiwan)*	*MSSA (28.6%)* *MRSA (28.6%)* Other (not specified) (21.4%) Negative culture (21.4%)	N.A.
Hunter et al. 2015 [5] *(USA)*	*MSSA (45%)* Negative culture (36%)	N.A.
Kaminski et al. 2007 [21] *(Germany)*	*MSSA (60%)* *Staphylococcus haemolyticus (20%)* *Staphylococcus intermedius (20%)*	Intra venous drug abuser (40%)
Kao et al. 2019 [1] *(Taiwan)*	*Staphylococcus (3.9%)* *MRSA (2.0%)* *Streptococcus species (2.0%)* *Escherichia coli (3.9%)* *Salmonella species (3.9%)* *Corynebacterium (2.0%)* Polymicrobial (3.9%) Negative culture (78.4%)	N.A.
Khazi et al. 2020 [22] *(USA)*	N.A.	N.A.
Kim et al. 2003 [23] *(South Korea)*	N.A.	N.A.
Kim et al. 2009 [4] *(South Korea)*	*MSSA (85%)* *Streptococcus pneumoniae (5%)* *Hemophilus influenzae (5%)* *Salmonella species (2%)* *Neisseria meningitidis (2%)* *Escherichia coli (2%)*	N.A.
Kim et al. 2018 [24] *(South Korea)*	*MSSA (42.9%)* *Streptococcus Agalactiae (14.2%)* Negative culture (42.9%)	N.A.
Kunze et al. 2020 [25] *(USA)*	*MSSA (14.3%)* *MRSA (9.4%)* *Coagulase-negative Staphylococcus aureus (23.8%)* *Serratia marcescens (4.8%)* *Pseudomonas aeruginosa (2.4%)* *Vancomycin-resistant Enterococcus (VRE) (2.4%)* *Group G Streptococcus (2.4%)* *Streptococcus viridans (4.8%)* Culture results undocumented in existing charts (11.9%) Negative culture from culturing tissue collected at stage 1 (26.2%)	N.A.
Lee et al. 2014 [27] *(South Korea)*	*MSSA (44.4%)* Negative culture (55.6%)	N.A.
Li et al. 2016 [28] *(China)*	*Mycobacterium tuberculosis (100%)*	Haematogenous (100%)
Lustig et al. 2007 [29] *(France)*	*Mycobacterium tubercolosis (47.1%)* *Staphylococcus aureus (52.9%)*	N.A.
Nusem et al. 2006 [30] *(Australia)*	*MSSA (66.6%)* Other (not specified) (16.7%) Negative culture (16.7%)	N.A.
Ohtsuru et al. 2016 [31] *(Japan)*	*MRSA (33.3%)* *MSSA (20.0%)* *MRSE (13.3%)* *Streptococcus agalactiae (6.7%)* *Enterococcus faecalis (6.7%)* *Bacteroides fragilis (6.7%)* *Mycobacterium tuberculosis (6.7%)* Negative culture (6.7%)	Incidence of infection at another location: 57.1% (group A); 40% (group B). Compromising factors (pyogenic cervical osteomyelitis or septic arthritis of the knee, removal of a foreign body from the buttocks, haemodialysis, diabetes, drainage of pus from recalcitrant pressure sores on the buttocks): 57.1% (group A); 100 (group B)
Papanna et al. 2017 [32] *(Japan)*	*MSSA (33.3%)* *MRSA (2.78%)* Other (not specified) (2.78%) Negative culture (61.1%)	N.A.
Park et al. 2005 [33] *(South Korea)*	*Mycobacterium tuberculosis (34.7%)* Pyogenic (not specified) (65.3%)	N.A.
Romanò et al. 2012 [41] *(Italy)*	*MSSA (50%)* *MRSA (20%)* Negative culture (20%) Others (not specified) (25%)	Haematogenous (42.1%) Post-operative (Post-osteosynthesis) (57.9%) After a local injection (5.3%)
Russo et al. 2021 [35] *(Italy)*	*MSSA (28%)* *MRSA (12%)* *Streptococcus species (4%)* *Pseudomonas species (8%)* *Mycobacterium species (8%)* *Escherichia coli (4%)* *Proteus species (4%)* Polymicrobial (8%) Negative culture (24%)	Post-operative (16%) Post-infiltrative (8%) Primary (76%)
Schroder et al. 2016 [36] *(Germany)*	*MSSA (28.2%)* Other (not specified) (43.8%) Negative culture (28.2%)	N.A.
Shen et al. 2013 [37] *(China)*	N.A.	N.A.
Xu et al. 2019 [38] *(China)*	*Coagulase-negative Staphylococcus (27.3%)* *MSSA (3.6%)* Resistant organism (not specified) (3.6%) Gram-negative organism (not specified) (10.9%) Polymicrobial (9.1%) Other organism (14.5%) Negative culture (30.9%)	Haematogenous (9.1%) Post-operative (69.1%) After a local injection (5.5%) Unknown (16.4%)
Yamamoto et al. 2001 [39] *(Japan)*	*MSSA (50.0%)* Other (not specified) (50.0%)	Steroidal drugs to treat a subarachnoid hemorrhage and thrombophlebitis of the leg (1 patient, 25%); Treatment for diabetes for 25 years (1 patient, 25%);
Yoo et al. 2009 [40] *(South Korea)*	N.A.	N.A.

*N.A.* not available; *MRSA* methicillin-resistant *Staphylococcus aureus; MSSA* methicillin-sensitive *Staphylococcus aureus*

Culture negative infections were reported to range from 16.7 to 78.4% [1,3,5,11,14,15,18,19,24–27,30,32,35,36,38]..

The cause of infection was clearly described in 10 papers included [3,12,18,21,28,31,35,38,39,41]. The rate of hematogenous infections ranged from 9.1% [38] to 65.3% [3], if we exclude the study by Li et al. [28] which described only tubercular SA with a 100% rate of hematogenous infections. Kaminski et al. [21] reported a 40% of patients using intravenous drugs, hence suggesting an hematogenous contagion way. Infections after surgery were identified, ranging from 16% [35] to 69% [38], even though acute or chronic onsets weren’t distinguished. Post-infiltrative septic arthritis was described in only 5 studies [3,18,35,38,41], with a rate varying from a 5% [41] to 14% [18] of treated cases.

Furthermore, Russo et al. [35] described that the 76% of septic arthritis were primary infections with a diagnosis based on one or a combination of clinical signs of infection, elevated serum C-reactive protein (CRP) and erythrocyte sedimentation rate ESR values, radiographic findings of bone resorption and/or loss of articular space, intra-operative purulence, and positive intra-operative and/or synovial fluid microbiology.

### Treatment options

Three main surgical options recommended for the treatment of septic arthritis such as arthroscopic debridement/lavage and one-stage or two-stage (either after resection arthroplasty or an antibiotic-loaded spacer implantation) total arthroplasties (Table 3) were described. Among the studies included in our systematic review 16 [1, 11–16, 18, 25, 26, 28, 32, 35, 37, 38, 41] reported a two-stage surgical treatment of the hip’s SA. In twelve papers [11, 13–16, 18, 25, 26, 28, 35, 37, 41] a two stage procedure was the only treatment evaluated, while in 3 studies [1,12,32] two-stage and one-stage procedures were considered. One stage procedure was performed in 446 adult patients affected by septic arthritis of native hip [1,4,20,23,29,33,40]. Proximal femur arthrotomies weren’t practiced as the only procedure in any of the articles included in this review but were part of one or two stage procedure valued in 6 studies [1,3,13,17,22,31] and utilized as a salvage operation by Anagnostakos et al. [11] and Park et al. [33] whenever the two or one-stage procedure failed. Arthroscopic and open debridement were the treatment option in 79 and 7 patients, respectively.

**Table 3:** Treatment options and clinical outcomes of Septic Arthritis of Hip

First author, year, Nation	No Hips	Treatment One stage vs two stage vs arthroscopy	Duration antibiotic treatment	Outcome (infection eradication rate)	Treatment failure
Anagnostakos et al. 2016 [11] *(Germany)*	22	Two stage	6 weeks	First stage 87% Second stage 100%	Girdlestone
Bauer et al. 2010 [12] *(France)*	22	Two stage (62%) One stage (38%)	80 days	85% 100%	
Chen et al. 2008 [13] *(China)*	28	Two stage (Gilderstone+THA)	4–6 weeks + 28 days (following arthroplasty)	86%	N.A.
Cho et al. 2018 [14] *(South Korea)*	10	Two stage	Positive culture: IV specific antibiotic. Negative culture: IV empirical 3 weeks therapy + oral	100%	N.A.
Choe et al. 2015 [15] *(Japan)*	27	Two stage	until the serum CRP decreased to less than 1 mg/dl or for maximum of 3 months.	100%	N.A.
El Ganzoury et al. 2015 [16] *(Egypt)*	23	Two stage	6 weeks intravenous	90%	Repeat spacer
Ferrand et al. 2016 [17] *(France)*	12	Arthroscopy (8%) Arthrotomy/washout (52%)	8 days intravenous and 52.5 oral.	N.A.	N.A.
Fleck et al. 2011 [18] *(USA)*	14	Two stage (10/14)	6 weeks	92.8% after one spacer 100% after two spacers	N.A.
Flores-Robles et al. 2019 [3] *(Spain)*	7	Two groups: one treated with initial medical therapy and one with initial surgical therapy: Arthroscopy Arthrotomy	30 days	N.A.	Surgery (at least 1 arthroscopy or arthrotomy) following failure of medical therapy
Fukushima et al. 2021 [19] *(Japan)*	5	Arthroscopy	3 weeks intravenous + 3 months oral	100%	N.A.
Gao et al. 2010 [20] *(China)*	19	One stage	Intraoperative	100%	N.A.
Huang et al. 2010 [26] *(Taiwan)*	15	Two stage	1 week intravenous	93.4% Only 1 patient (6.6%) failure attributed to an immunocompromised status due to alcoholism and heroin abuse) Second attempt: 100%	Additional debridement with reinsertion of a new spacer and a second 7- day course of intravenous antibiotic therapy before THA.
Hunter et al. 2015 [5] *(USA)*	3	Open debridement (68%) Arthroscopy (32%)	3 to 12 weeks of oral or intravenous therapy, determined by infectious disease consultants	62%	additional surgical debridement
Kaminski et al. 2007 [21] *(Germany)*	5	fenestration Arthroscopy	4 weeks	100%	N.A.
Kao et al. 2019 [1] *(Taiwan)*	51	One stage 53% Two stage 27%	85 days	74% resection arthroplasty (one stage) 92.9% revision THA (two stages)	Surgical debridement for 5 patient with recurrent hip infections within 2 years after surgery.
Khazi et al. 2020 [22] *(USA)*	421	Arthroscopy (8%) Arthrotomy (92%)	N.A.	100%	N.A.
Kim et al. 2003 [23] *(South Korea)*	170	One stage	N.A.	99.5%	N.A.
Kim et al. 2009 [4] *(South Korea)*	62	One stage	2 days (primary THA)	98%	Revision surgery
Kim et al. 2018 [24] *(South Korea)*	7	Arthroscopy	4–6 weeks if negative culture 4–7 weeks specific if positive culture	100%	N.A.
Kunze et al. 2020 [25] *(USA)*	12	Two stage	Intravenous for 6 weeks	91.7%	Spacer exchange
Lee et al. 2014 [27] *(South Korea)*	9	Arthroscopy	4–6 weeks	88.8% after first arthroscopy 100% after second arthroscopy	N.A.
Li et al. 2016 [28] *(China)*	9	Two stage 44% spacer implantation 56% debridement	> 12 months after the first operation.	100%	N.A.
Lustig et al. 2007 [29] *(France)*	17	One stage	N.A.	94%	N.A.
Nusem et al. 2006 [30] *(Australia)*	6	Arthroscopy	3 weeks intravenous + 3 weeks oral	100%	N.A.
Ohtsuru et al. 2016 [31] *(Japan)*	15	Arthroscopy, debridement, resection arthroplasty, spacer;	4 weeks intravenous + 2 weeks oral	66.7%	musculocutaneous flap transposition
Papanna et al. 2017 [32] *(UK)*	18	Two stage 61% One stage 39%	N.A.	100% 100%	N.A.
Park et al. 2005 [33] *(South Korea)*	75	One stage	N.A.	98.7%	Girdlestone
Romanò et al. 2012 [41] *(Italy)*	20	Two stage	4–6 weeks	95%	N.A.
Russo et al. 2021 [35] *(Italy)*	25	Two stage	2 weeks intravenous + 4 weeks oral/ targeted intravenous	100%	N.A.
Schroder et al. 2016 [36] *(Germany)*	7	Arthroscopy	4 weeks	100%	N.A.
Shen et al. 2013 [37] *(China)*	5	Two stage	6 weeks	100%	N.A.
Xu et al. 2019 [38] *(China)*	55	Two Stage	> 4 weeks	93%	N.A.
Yamamoto et al. 2001 [39] *(Japan)*	4	Arthroscopy	2 weeks oral	100%	N.A.
Yoo et al. 2009 [40] *(South Korea)*	38	One stage	N.A.	97%	N.A.

*N.A.* Not Available

As for antibiotic therapy protocols, 7 studies [20,22,23,29,32,33,40] didn’t mention what therapy had been conducted during the treatment of SA and for how long.

The duration of antibiotic therapy consisted in a from 4 to 6 weeks antibiotic protocol in 18 papers [3, 11, 13, 16, 18, 19, 21, 24, 25, 27, 30, 31, 35–38, 41]. Three papers presented shorter than 4-weeks antibiotic protocols [4,26,39, 6] [1,5,12,14,15,28] practiced instead a longer antibiotic regimen (> 6 weeks).

Successful treatment of SA, defined as infection eradication rate after antibiotic discontinuation, was reported in 32 papers included in the systematic review ranging from 62% [5] to 100% of patients.

Two-stage procedures have reported a high eradication rate following the second-step surgery, ranging from 85% [12] to 100% [11,14,15,28,32,35,37].

Only six studies [4,20,23,29,33,40] reported patients treated exclusively by one-stage revision arthroplasties with an eradication rate ranging from 94% [29] to 100% [20].

Although 12 studies [3,5,17,19,21,22,24,27,30,31,36,39] included arthroscopic debridement in their research, only 7 [19,21,24,27,30,36,39] regarded cases treated exclusively through arthroscopy. The infection eradication rate after hip arthroscopic debridement/lavage was reported to be of 100% of treated cases in 6 out of 7 studies included in this review, with the only exception of the article by Lee et al. [27] in which 8 out of 9 patients who underwent arthroscopy healed from infection, whilst 1 patient reached eradication after a second arthroscopic procedure.

The management of failed patients that experienced a persistent infection varied among the studies. Only 9 of the 36 articles included in this review described their management of failed cases (Table 3).

Timing from diagnosis of septic arthritis to surgical procedure varied across the valued papers: 5 of 34 papers mentioned this parameter. Anagnostakos et al. [11] diagnosed infection between 4 weeks and 6 months prior to surgery, while Romanò et al. [41] between 6 and 9 months. Yamamoto et al. [39] and Fukushima et al. [19] treated arthroscopically the patients included in each study, 36 days after diagnosis and “immediately after diagnosis”, respectively. Ohtsuru et al. [31] studied two different cohorts of patients: the first group averaged 10 days from diagnosis of septic arthritis of the hip and surgical treatment, whilst the second group averaged a 95-days interval.

## Discussion

Septic Arthritis of the hip is a disease with a relative low incidence [2] but causes pain and disability to the affected patients with a mortality rate estimated to hover around 10%. Methicillin sensitive *Staphylococcus aureus* appears to be the most common causative agent for septic arthritis of the hip. The culture negative infections occur in a percentage that varies from 16.7 to 78.4% of the cases [1,3,5,11,14,15,18,19,24–27,30,32,35,36,38].

The treatment of hip infection in adult patients is influenced by several factors, but the choice of the best option depends on the type of infection (active or quiescent). Various surgical treatment options are currently available for the orthopedic surgeon who faces a SA such as arthroscopic lavage/debridement, resection arthroplasty (arthrotomy) and Total Hip Replacement (THR) in one or two stages.

The chosen treatment wasn’t influenced by the age of the patients in any of the reviewed articles, but, noticeably, Nusem et al. [30] treated exclusively with arthroscopy the youngest cohort of patients among all papers.

Arthroscopy is usually effective to remove infective materials and to debride necrotic tissues. Although Flores-Robles et al. [3] highlighted that the arthroscopic debridement of the hip SA reported a lower recurrence of infection than conservative approach, more than one procedure is often mandatory to resolve the infection process [27].

The resection arthroplasty as described by Girdlestone in 1943 may be effective on eradicating the infection, but the sequelae include chronic limp, length discrepancy, and only partial pain relief, even though the procedure itself has been vastly modified over the years [35]. One and two-stage THR, whether the first step was constituted by a resection arthroplasty or the implantation of an antibiotic-loaded hip spacer, have proven to be very effective on eradicating infection and have excellent long term functional outcomes [6,7,14,26], but require consistent technical skill to face the deformities caused by the SA (deformation of the acetabulum, insufficient bone stock in the superolateral acetabulum leading to insufficient coverage of the cup, and abnormal positioning of the hip contributing to accelerated aseptic loosening, etc.) [40] and the resources to support potential longer hospital stays and higher costs for implants [1].

Chen et al. [13] reported on a 28-hips population treated with a Girdlestone arthrotomy followed by a THR, with an average follow up of 77 months and a rate of eradication for infection of 86%, suggesting that implanting an antibiotic-loaded spacer may help to improve the microbiological efficacy of the treatment. In the study by Choe et al. [15] the two-stage procedure was applied to 27 patients suffering from both SA and PJI, with similar functional outcomes and a full 100% of free-from-infection (defined as serum CRP decreased to less than 1 mg/dl or for maximum of 3 months) patients at a 33 and 38-months period, respectively. Li et al. [28] reported a 100% eradication rate from tubercular SA treating patients with either spacer implantation or extensive debridement alone during a first surgical step, preceded by 2 weeks of antitubercular chemotherapy and followed by for at least 3 months of the same pharmacological protocol, plus 9 months after the THA for a total of 12 months. One stage treatment showed equal if not higher infection eradication rates (85% vs 100% according to Bauer et al. [12] on 22 cases with 60 months follow-up) with correct diagnostic work-up to treatment and timing.

Recently, the second international consensus meeting on peri-prosthetic joint infection tried to standardize the treatment of the patient affected by septic arthritis differentiating between active and quiescent local infective process of the hip or knee. Patients affected by quiescent SA present a history of infection with no clinical, laboratory and radiological signs of local active infection.

One-stage arthroplasty is recommended for quiescent infections instead of two-stage arthroplasty that is indicated in those patients affected by active infections at the time of arthroplasty [8]. The success rate seems to be quite similar between one and two stage when performed in patients affected by quiescent and active infection, respectively. This study has a few drawbacks. First, this systematic review was performed on level II or level IV small case series. Moreover, the lack of standardization between papers regarding the joint damage, host, pathogen and diverse techniques may have contributed to heterogeneity between studies. This limitation prevented us to compare techniques especially for the infection eradication rate.

## Conclusion

The evidence emerged from this review suggests that *Staphylococcus aureus* is the most common microorganism isolated followed by culture negative infections. The specific pathogen responsible for a given infection, including negative cultures, wasn’t a criteria for the selection of the surgical option, but rather it modified the antibiotic protocol followed by each patient. Arthroscopic, one and two stage procedures can be effective in the treatment of hip septic arthritis taking in consideration the type of infection. However, further perspective studies would be needed to establish an algorithm of treatment options.

## Data Availability

Not applicable.

## Abbreviations

CRP: C-Reactive Protein
ESR: Erythrocyte Sedimentation Rate
N.A: Not Available
SA: Septic Arthritis
PJI: Peri-prosthetic Joint Infection
THR: Total Hip Replacement
MRSA: Methicillin-Resistant *Staphylococcus aureus*
MSSA: Methicillin-sensitive *Staphylococcus aureus*
MRSE: Methicillin-Resistant *Staphylococcus epidermidis*
